# A Case of Immune Checkpoint Inhibitor-Induced Probable Myocarditis and Treatment Response

**DOI:** 10.7759/cureus.39692

**Published:** 2023-05-30

**Authors:** Mubariz A Hassan, Yashvardhan Batta, Muhammad Adil Afzal

**Affiliations:** 1 Internal Medicine, Howard University Hospital, Washington DC, USA; 2 Internal Medicine, Howard University College of Medicine, Washington DC, USA; 3 Internal Medicine, St. Joseph's Regional Medical Center, Paterson, USA

**Keywords:** abnormal cardiac enzymes, systemic steroids, drug-induced myocarditis, myocardial injury, immune check-point inhibitor

## Abstract

Immune checkpoint inhibitors (ICI) are a new class of pharmaceuticals that facilitate the immune system in identifying and targeting cancerous cells. However, suppressing immune regulation can often cause immune-mediated adverse events. One such downstream effect recently recognized is ICI-associated myocarditis. This case involves a 67-year-old female patient with a medical history of metastatic small-cell lung carcinoma undergoing chemotherapy with atezolizumab (third cycle) and the carboplatin-etoposide regimen (fourth cycle). The patient presented to the medical service with chest discomfort and fatigue. Elevated cardiac markers were observed, despite the absence of ischemic changes on electrocardiography and patent coronary arteries on cardiac catheterization. Cardiac magnetic resonance imaging (MRI) did not reveal any significant fibrosis in the cardiac muscle; however, an endomyocardial biopsy noted mild fibrosis. Corticosteroid treatment resulted in the normalization of cardiac enzyme levels and subsequent symptom resolution. ICI-associated myocarditis typically manifests within two months of initiating therapy. However, this case report spotlights the occurrence of a milder form of myocarditis after three months of ICI treatment.

## Introduction

Immune checkpoint inhibitors (ICI) have revolutionized chemotherapeutic management by harnessing the power of the immune system to combat malignancies. These monoclonal antibodies specifically target and inhibit negative immune regulation receptors expressed on immune cells, such as cytotoxic T-lymphocyte-associated protein 4 (CTLA-4), programmed cell death protein 1 (PD-1), and programmed death-ligand 1 (PD-L1) [1]. By inhibiting these checkpoints, ICI unleashes the immune system's ability to recognize and attack cancer cells. While the advantages of ICI therapy seem evident, it is imperative to consider the emerging concerns of immune-mediated adverse effects, such as myocarditis. The underlying mechanisms of ICI-associated myocarditis have not yet been fully elucidated, but several working hypotheses have been proposed. One hypothesis postulates that ICI-associated myocarditis arises from myocardial T-cell infiltration and inflammation, akin to the pathophysiological immune response of viral myocarditis. Activated T-cells may release pro-inflammatory cytokines, including interferon-gamma and tumor necrosis factor-alpha, thereby further contributing to myocardial injury.

ICI-associated myocarditis can range from mild symptoms to severe cardiac dysfunction, often presenting with chest discomfort, fatigue, shortness of breath, or palpitations. Elevated cardiac enzymes indicate myocardial damage, and in severe cases, it can progress to heart failure and end-organ damage. Diagnostic evaluations include electrocardiography, cardiac biomarker testing, echocardiography, and cardiac MRI. Managing ICI-associated myocarditis requires a multidisciplinary approach, involving prompt diagnosis, ICI discontinuation, immunosuppressive agent administration, and close monitoring of cardiac function. Advanced interventions like mechanical circulatory support or immune-modulating therapies may be necessary in certain cases.

## Case presentation

A 67-year-old female with a past medical history significant for small cell lung carcinoma on atezolizumab (third cycle), carboplatin, and etoposide regimen (fourth cycle) with metastasis to the liver and chest wall, hypertension, hypothyroidism, and remote smoking history presented with complaints of reduced oral intake, failure to thrive, and generalized weakness over the past two weeks. She also reported central chest discomfort and worsening dyspnea with minimal exertion. A review of systems was negative for orthopnea, paroxysmal nocturnal dyspnea, lower extremity swelling, nausea, fever, cough, or syncope.

On examination, the patient appeared diaphoretic with a temperature of 36.4°C, heart rate of 92 beats per minute, blood pressure of 104/67 mmHg, respiratory rate of 18 breaths per minute, and oxygen saturation of 94% on room air. A physical examination was performed and it was unremarkable for any pertinent findings. For possible sepsis concerns, early investigations and lab work were performed, including blood cultures and initiation of broad-spectrum antibiotics. However, the septic workup returned negative. A viral panel was also performed that also came back negative. The outpatient oncologist was informed, and considering the patient's clinical situation, myositis and myocarditis were considered differentials.

**Figure 1:**
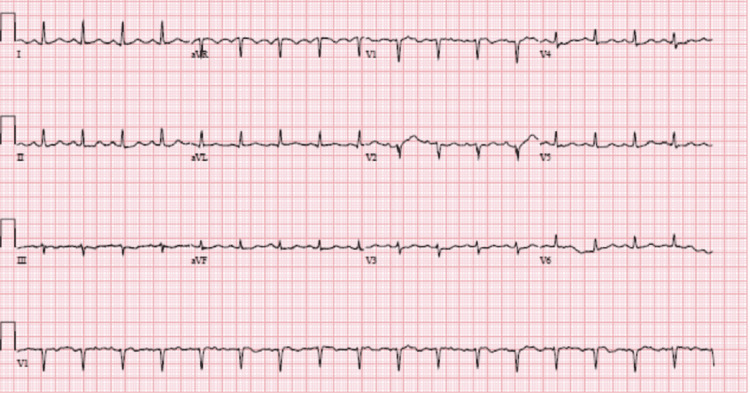
ECG showing normal sinus rhythm with sinus tachycardia with no ischemic changes ECG: Electrocardiogram

A cardiac workup revealed elevated cardiac enzymes as shown in Table 1. An electrocardiogram (EKG) showed sinus tachycardia with low voltage Figure 1. Echocardiography demonstrated hypokinesia of the anterior and inferior septum and inferolateral wall, with an estimated ejection fraction of 40-45% likely secondary to persistent tachycardia during the study as seen in Video 1.

**Table 1:** Laboratory results

Basic Labs	Results	Reference Range
White Blood Cells	10.5	3.2-10.6x10^9^/L
Hemoglobin	12.1	14.6-17.8 g/dL
Hematocrit	29.5	40.8-51.9 %
Platelets	316	177-406x10^9^/L
Sodium	140	135-145 mEq/L
Chloride	99	95-111 mEq/L
Blood Urea Nitrogen	20	7-25 mg/dL
Creatinine	1.5	0.6-1.2 mg/dL
Potassium	4.6	3.5-5.1 mEq//L
Magnesium	1.94	1.7-2.5 mg/dL
BNP	4472	<100 Pg/mL
Troponin (trend over 2 days)	0.22>0.19>0.07>0.05>0.04	<0.03 Ng/mL

**Video 1:** Echocardiogram showing Wall motion abnormality as seen on Apical 3 Chamber View.

ICI was discontinued, and the patient was initiated on corticosteroids. Cardiac MRI did not show any late gadolinium enhancement suggestive of scars or fibrosis, and the overall findings did not meet the Lake Louise Criteria for myocarditis. Cardiac catheterization performed during the hospital stay revealed patient coronaries with mild disease in the right coronary artery. An endomyocardial biopsy showed areas of fibrosis but no evidence of myocarditis. Given patient symptoms, wall motion abnormalities on cardiac echocardiogram and positive cardiac biomarkers, a working diagnosis of probable myocarditis was made as per definition criteria. The patient demonstrated clinical improvement with steroid therapy, and repeat cardiac enzymes normalized. The final diagnosis of a mild case of ICI-induced probable myocarditis was made. The patient was discharged home on a steroid taper and instructed to follow up with Cardiology and Oncology as outpatient care.

## Discussion

Immune checkpoint inhibitors (ICIs) are a class of drugs that work by targeting specific proteins in immune cells or cancer cells, allowing the immune system to recognize and attack cancer cells more effectively. One of the key mechanisms of ICIs involves blocking the interactions between immune checkpoint proteins, such as PD-1 and CTLA-4, and their corresponding ligands. These interactions normally serve as "checkpoints" that prevent immune cells from attacking healthy cells, including cancer cells. By inhibiting these interactions, ICIs essentially release the "brakes" on the immune system, unleashing its full potential to recognize and destroy cancer cells. This mechanism enhances the immune response against tumors and has shown remarkable success in treating various types of cancer. However, it is important to note that the activation of the immune system by ICIs can also lead to immune-related adverse events, which require careful monitoring and management. ICIs have revolutionized the field of cancer therapy, significantly improving overall survival and outcomes for patients with various cancer types [2-4]. However, the use of ICIs is not without risks. One of the significant side effects associated with ICIs is immune-mediated adverse events, which can affect multiple organ systems, including the heart [5].

In this case, a 67-year-old female with a history of metastatic small-cell lung carcinoma on chemotherapy with atezolizumab presented with symptoms of chest discomfort and fatigue. A cardiac workup showed elevated cardiac markers, hypokinesia of the anterior and inferior septum and inferolateral wall on the echocardiogram, and fibrosis of cardiac muscles on endomyocardial biopsy with no evidence of myocarditis. The patient was diagnosed with ICI-induced probable myocarditis and was treated with steroid therapy resulting in the normalization of cardiac enzymes and resolution of symptoms.

The clinical presentation of ICI-induced myocarditis can range from mild to severe, with symptoms typically appearing within one to two months of initiating therapy. However, myocarditis can also manifest even after several months of ICI therapy [6]. The mechanism underlying ICI-induced myocarditis is not fully understood, but it is thought to be related to T-cell infiltration of the myocardium, similar to viral myocarditis [6,7]. This can lead to a spectrum of clinical presentations, ranging from asymptomatic elevation in cardiac enzymes to severe decompensation with end-organ damage. Given the potential severity of this adverse event, clinicians need to maintain a high index of suspicion for myocarditis in patients receiving ICIs and presenting with cardiac symptoms [8,9].

## Conclusions

In conclusion, while ICIs offer significant benefits in immune-modulated chemotherapy, they also carry the risk of immune-mediated adverse events, including myocarditis. This case highlights the need for clinicians to be aware of the potential for ICI-induced myocarditis and to consider this diagnosis in patients presenting with cardiac symptoms who have received ICI therapy. Early recognition and prompt treatment with steroids can lead to improved outcomes for these patients. Further research is needed to better understand the underlying mechanisms of ICI-induced myocarditis and to develop strategies for prevention and treatment.
